# Inflammatory cytokine levels in multiple system atrophy: A protocol for systematic review and meta-analysis

**DOI:** 10.1097/MD.0000000000021509

**Published:** 2020-07-31

**Authors:** HongZhou Wang, WanHua Wang, ZhongQuan Yi, PanWen Zhao, Hui Zhang, PingLei Pan

**Affiliations:** a Department of Neurology, Kunshan Hospital, Affiliated to Jiangsu University, Kunshan; b Department of Central Laboratory, Yancheng School of Clinical Medicine of Nanjing Medical University, Yancheng; c Department of Neurology and Department of Central Laboratory, Yancheng School of Clinical Medicine of Nanjing Medical University, Yancheng, PR China.

**Keywords:** biological marker, cytokine, inflammation, meta-analysis, multiple system atrophy

## Abstract

**Background:**

Multiple system atrophy (MSA) is a fatal neurodegenerative disease that progresses very rapidly and has a poor prognosis. Some studies indicate that the level of inflammatory cytokines may be related to MSA. However, no consistent conclusion has been drawn yet. The purpose of our research is to perform a meta-analysis to investigate whether the level of inflammatory cytokines is altered in MSA.

**Methods:**

Case-control studies on inflammatory cytokine levels in MSA will be searched in the following 3 databases: PubMed, Embase, and Web of Science from the database start time to March 17, 2020. Two independent authors will conduct research selection, data extraction, and quality evaluation. Data synthesis, subgroup analysis, sensitivity analysis, and the meta-analysis will be performed using Stata15.0 software.

**Results:**

This study will provide a comprehensive review of all studies on inflammatory cytokine levels in MSA.

**Conclusion:**

To the best of our knowledge, this study will be the first meta-analysis that provides the quantitative evidence of inflammatory cytokine levels in MSA.

**Registration Number:**

INPLASY202060034.

## 1 Introduction

Multiple system atrophy (MSA) is a fast progressive and fatal neurodegenerative disorder that is characterized clinically by autonomic failure, cerebellar ataxia, and parkinsonism in various combinations.^[1,2]^ There are 2 main subtypes based on the clinical characteristics of MSA:

(1) the MSA-P subtype with Parkinson's syndrome as the prominent manifestation and
(2) the MSA-C subtype with the cerebellar ataxia symptoms as the prominent manifestations.^[3,4]^

The prognosis of MSA is poor, with an average life span of 6 to 9 years from morbidity to death according to a report.^[5–7]^ So far, there is only limited symptomatic treatment, and no effective drugs have been found to cure MSA.^[8]^ Therefore, it is necessary to find biomarkers to help better understand the pathophysiology of MSA.

A great number of glial cytoplasmic inclusions in the oligodendroglia cytoplasm are a pathological hallmark of MSA.^[9]^ Many studies have found that there are a greater number of activated microglia in MSA cases.^[10,11]^ Potential toxic products include inflammatory cytokines and other inflammatory markers, produced and released by activated microglia.^[12,13]^ Therefore, it is speculated that there is an inflammatory state in the brain of MSA, which may be associated with the neurophysiological cause of the disease.^[14]^

To date, some studies have used easily accessible and less invasive peripheral blood to measure inflammatory cytokine levels in patients with MSA.^[15–18]^ At the same time, some studies have used cerebrospinal fluid to detect cytokine levels in patients with MSA, because cerebrospinal fluid specimens are closely related to the brain and are not affected by drugs such as nonsteroidal anti-stimulation drugs.^[19–23]^ Although some studies have evaluated inflammatory cytokines in MSA, the results are not always consistent.^[15–23]^ Hall et al^[23]^ found IL-8 is increased in patients with MSA compared to healthy controls. While Rydbirk et al^[22]^ found no difference of IL-8 between patients with MSA and healthy controls. The results of individual studies can be quantitatively combined using meta-analysis techniques to increase the strength of the evidence. Thus, we will perform a meta-analysis to study the concentration of inflammatory cytokines in the peripheral blood and cerebrospinal fluid specimens of MSA patients.

## 2 Methods

In this systematic review and meta-analysis, the principles of the Preferred Reporting Items for Systematic reviews and Meta-Analyses checklist will be fully followed.^[24]^ This protocol has been registered on the International Platform of Registered Systematic Review and Meta-Analysis Protocols (INPLASY) in June 2020, and its registration number is INPLASY202060034 (URL = https://inplasy.com/inplasy-2020-6-0034/).

### 2.1 Ethics approval

Because the data used in this paper are from published studies without the involvement of individual or animals’ experiments, the ethical approval is not required.

### 2.2 Eligibility criteria for study selection

#### 2.2.1 Types of studies

We will choose case-control studies that compare the levels of cytokines between MSA patients and healthy controls. Inclusion criteria consisted of:

(1) study design limited to case-control studies,
(2) studies measuring peripheral blood or CSF inflammatory factor concentrations;
(3) the inclusion of healthy subjects as controls.

Exclusion criteria included:

(1) Non-human studies, reviews, conference abstracts, editorials, or letters will be excluded unrelated to the research topic;
(2) case reports and case series;
(3) without healthy controls;
(4) without necessary data;
(5) the samples were collected before patients were diagnosed with MSA.

#### 2.2.2 Types of participants

Patients (aged over 18 years) diagnosed with MSA will be included in the study. Patients with other serious complications, a history of brain surgery, or other serious neurodegenerative diseases will be excluded from this study.

#### 2.2.3 Types of interventions

We will mainly study the differences in the level of inflammatory cytokines in cerebrospinal fluid or peripheral blood between MSA patients and healthy controls.

#### 2.2.4 Type of comparators

We will choose healthy controls, who has no disease.

#### 2.2.5 Types of outcome measures

Main results: Differences in the concentration of inflammatory cytokines in peripheral blood or cerebrospinal fluid between patients with MSA and healthy controls.

### 2.3 Search methods in the study

Two independent authors will search the following databases: PubMed, Embase, and Web of Science. The search strategy will include the following phrases: ((Atrophy, Multiple System) OR (Multiple System Atrophies) OR (Multisystemic Atrophy) OR (Atrophies, Multisystemic) OR (Atrophy, Multisystemic) OR (Multisystemic Atrophies) OR (Multiple System Atrophy Syndrome) OR (Multisystem Atrophy) OR (Atrophies, Multisystem) OR (Atrophy, Multisystem) OR (Multisystem Atrophies) OR (Multiple System Atrophy) OR MSA) AND (inflammation OR cytokine OR chemokine OR interferon OR interleukin OR (transforming growth factor) OR (tumor necrosis factor) OR (C-reactive protein)). The search deadline is: March 17, 2020. The search strategy in the PubMed, Embase, and Web of Science databases are shown in Tables 1–3.

**Table 1:**
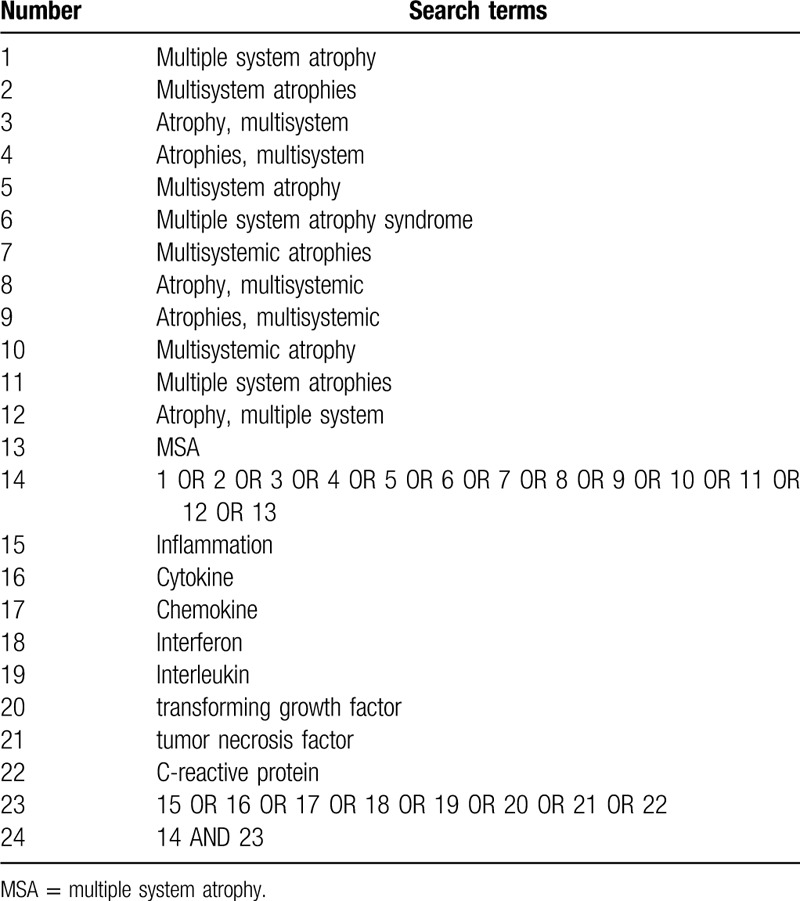
Search strategy for the PubMed database.

**Table 2:**
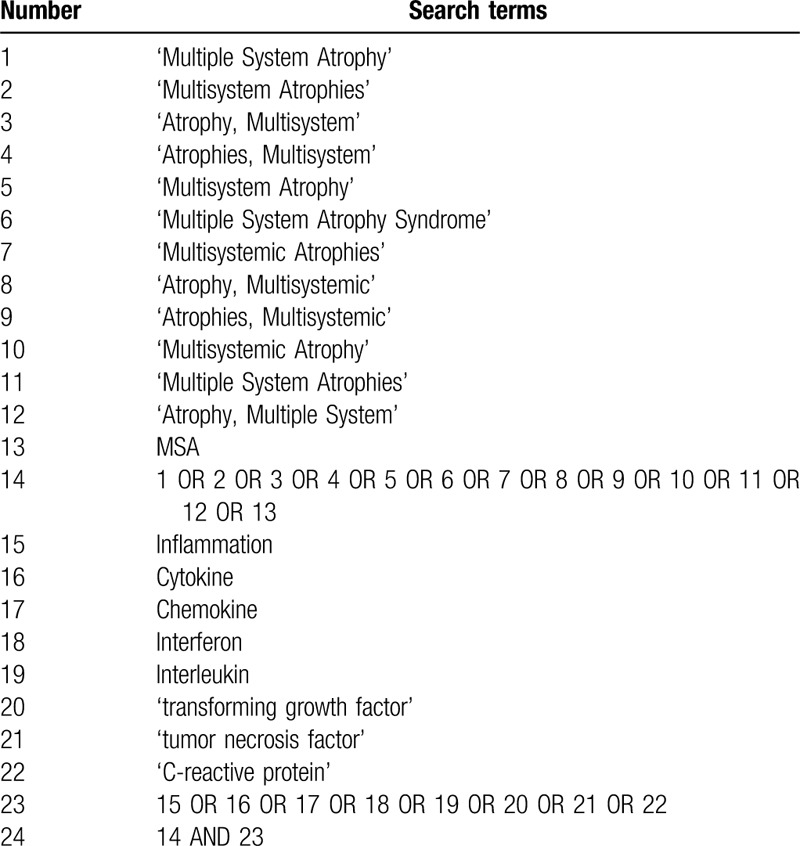
Search strategy for the Embase database.

**Table 3:**
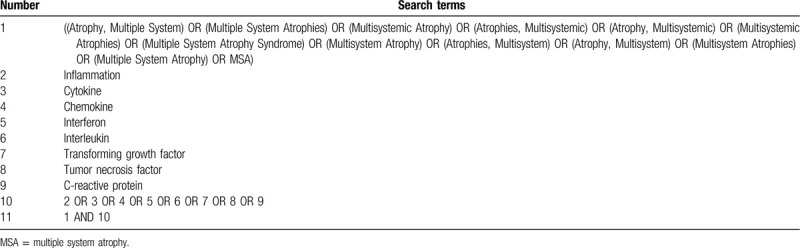
Search strategy for the Web of Science database.

### 2.4 Data collection

#### 2.4.1 Selection of studies

**Figure 1:**
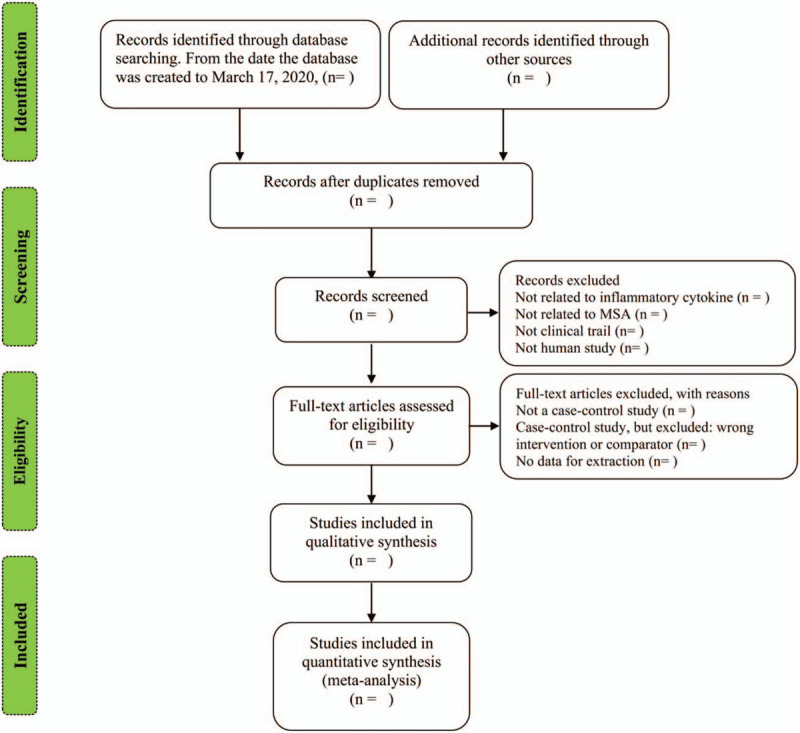
Flow diagram of study selection process.

The results of the above 3 database searches will be managed using endnote software (X7 version). Firstly, we will remove duplicate articles with the same author, title, and abstract (the same article is displayed in different databases). Secondly, we will screen the title and abstract of the article by 2 independent reviewers, select the studies that may meet the conditions, and exclude the studies that do not meet the selection criteria. Last but not least, we will filter the full text of the download. When the 2 reviewers disagree, we will discuss to solve it. If there are still objections, the third reviewer will analyze them. The reasons for the excluded articles will be recorded. The study selection process will be presented in the following Preferred Reporting Items for Systematic reviews and Meta-Analyses flow diagram (Fig. 1).

#### 2.4.2 Date extraction

We will extract the required data into Excel according to the established data extraction protocol. The contents collected will include: first author's name, year of publication, participants’ country, age, number of patients and controls, female: male ratio, duration of disease, race, the mean and the standard deviation of cytokine concentration for patients with MSA and controls, United MSA Rating Scale. One reviewer will extract all the data, and another reviewer will independently verify the extraction method.

#### 2.4.3 Risk of bias in included studies

Two independent reviewers will independently assess the methodological quality of each included study. We will use the Newcastle-Ottawa scale checklist.^[25]^ The range of this tool is from 0 to 9, which is from lowest quality to best quality. If there is a disagreement between the 2 reviewers, the issue will be resolved through discussion with the third reviewer.

### 2.5 Date analysis

We will use Stata 15.0 software to process and analyze the collected data. The *Q* test and *I*^2^ will be used to test the heterogeneity of the study. When the heterogeneity is relatively high (*I*^2^ > 50%), we will use a random-effects model to analyze the data; otherwise we will use a fixed-effects model.

### 2.6 Subgroup analysis

If the heterogeneity of the results is high and the data are sufficient, we will perform a subgroup analysis on the data in order to find the cause of the large heterogeneity. We will divide MSA patients into MSA-C and MSA-P subtypes according to clinical symptoms. At the same time, we will also analyze the race, nationality, duration of disease and other aspects of the subgroup analysis.

### 2.7 Sensitivity analysis

In order to ensure the robustness and reliability of the results, sensitivity analysis will be conducted by excluding highly biased studies.

### 2.8 Publication biases

To assess potential publication bias, if there is enough research, we will use funnel plot and Egger test.

## 3 Discussion

MSA is a very serious disease with a poor prognosis, so it is necessary to find effective biomarkers for diagnosing MSA. Previous clinical studies have reported the relationship between inflammatory cytokines and patients with MSA.^[15–23]^ However, there is no systematic comprehensive review and meta-analysis to discuss their relationship.

This article will analyze the concentration of cytokines in the peripheral blood and cerebrospinal fluid in MSA patients. We hope to find biomarkers that provide a basis for auxiliary diagnosis of MSA.

## Author contributions

**Conceptualization:** Hui Zhang, PingLei Pan

**Data curation:** ZhongQuan Yi, PanWen Zhao

**Formal analysis:** WanHua Wang, PanWen Zhao

**Investigation:** HongZhou Wang

**Methodology:** WanHua Wang, ZhongQuan Yi, PanWen Zhao,

**Software:** ZhongQuan Yi, PanWen Zhao

**Supervision:** PingLei Pan

**Validation:** Hui Zhang

**Writing – original draft:** HongZhou Wang

**Writing – review & editing:** Hui Zhang, PingLei Pan

## References

[1] Krismer F Wenning GK Multiple system atrophy: insights into a rare and debilitating movement disorder. Nat Rev Neurol 2017;13:232–43. 28303913 10.1038/nrneurol.2017.26

[2] Gilman S Wenning GK Low PA Second consensus statement on the diagnosis of multiple system atrophy. Neurology 2008;71:670–6. 18725592 10.1212/01.wnl.0000324625.00404.15 PMC2676993

[3] Pfeiffer RF Multiple system atrophy. Handb Clin Neurol 2007;84:305–26. 18808955 10.1016/S0072-9752(07)84046-2

[4] Ubhi K Low P Masliah E Multiple system atrophy: a clinical and neuropathological perspective. Trends Neurosci 2011;34:581–90. 21962754 10.1016/j.tins.2011.08.003 PMC3200496

[5] Ben-Shlomo Y Wenning GK Tison F Survival of patients with pathologically proven multiple system atrophy: a meta-analysis. Neurology 1997;48:384–93. 9040727 10.1212/wnl.48.2.384

[6] Schrag A Wenning GK Quinn N Survival in multiple system atrophy. Mov Disord 2008;23:294–6. 18044727 10.1002/mds.21839

[7] Figueroa JJ Singer W Parsaik A Multiple system atrophy: prognostic indicators of survival. Mov Disord 2014;29:1151–7. 24909319 10.1002/mds.25927 PMC4139446

[8] Mészáros L Hoffmann A Wihan J Current symptomatic and disease-modifying treatments in multiple system atrophy. Int J Mol Sci 2020;21:2775. 10.3390/ijms21082775 PMC7215736 32316335

[9] Papp MI Kahn JE Lantos PL Glial cytoplasmic inclusions in the CNS of patients with multiple system atrophy (striatonigral degeneration, olivopontocerebellar atrophy and Shy-Drager syndrome). J Neurol Sci 1989;94:79–100. 2559165 10.1016/0022-510x(89)90219-0

[10] Sakurai A Okamoto K Yaguchi M Pathology of the inferior olivary nucleus in patients with multiple system atrophy. Acta Neuropathol 2002;103:550–4. 12012086 10.1007/s00401-001-0500-x

[11] Williams GP Marmion DJ Schonhoff AM T cell infiltration in both human multiple system atrophy and a novel mouse model of the disease. Acta Neuropathol 2020;139:855–74. 31993745 10.1007/s00401-020-02126-w PMC7181566

[12] Wyss-Coray T Mucke L Inflammation in neurodegenerative disease--a double-edged sword. Neuron 2002;35:419–32. 12165466 10.1016/s0896-6273(02)00794-8

[13] Hanisch UK Microglia as a source and target of cytokines. Glia 2002;40:140–55. 12379902 10.1002/glia.10161

[14] Infante J Llorca J Berciano J Interleukin-8, intercellular adhesion molecule-1 and tumour necrosis factor-alpha gene polymorphisms and the risk for multiple system atrophy. J Neurol Sci 2005;228:11–3. 15607204 10.1016/j.jns.2004.09.023

[15] Brodacki B Staszewski J Toczyłowska B Serum interleukin (IL-2, IL-10, IL-6, IL-4), TNFalpha, and INFgamma concentrations are elevated in patients with atypical and idiopathic parkinsonism. Neurosci Lett 2008;441:158–62. 18582534 10.1016/j.neulet.2008.06.040

[16] Kaufman E Hall S Surova Y Proinflammatory cytokines are elevated in serum of patients with multiple system atrophy. PLoS One 2013;8:e62354. 23626805 10.1371/journal.pone.0062354 PMC3633844

[17] Csencsits-Smith K Suescun J Li K Serum lymphocyte-associated cytokine concentrations change more rapidly over time in multiple system atrophy compared to Parkinson disease. Neuroimmunomodulation 2016;23:301–8. 28395279 10.1159/000460297 PMC5491391

[18] Chen D Wei X Zou J Contra-directional expression of serum homocysteine and uric acid as important biomarkers of multiple system atrophy severity: a cross-sectional study. Front Cell Neurosci 2015;9:247. 26217177 10.3389/fncel.2015.00247 PMC4492156

[19] Santaella A Kuiperij HB van Rumund A Inflammation biomarker discovery in Parkinson's disease and atypical parkinsonisms. BMC Neurol 2020;20:26. 31952511 10.1186/s12883-020-1608-8 PMC6967088

[20] Compta Y Dias SP Giraldo DM Cerebrospinal fluid cytokines in multiple system atrophy: a cross-sectional Catalan MSA registry study. Parkinsonism Relat Disord 2019;65:3–12. 31178335 10.1016/j.parkreldis.2019.05.040

[21] Masuda T Itoh J Koide T Transforming growth factor-β1 in the cerebrospinal fluid of patients with distinct neurodegenerative diseases. J Clin Neurosci 2017;35:47–9. 27756506 10.1016/j.jocn.2016.09.018

[22] Rydbirk R Elfving B Andersen MD Cytokine profiling in the prefrontal cortex of Parkinson's Disease and Multiple System Atrophy patients. Neurobiol Dis 2017;106:269–78. 28732710 10.1016/j.nbd.2017.07.014

[23] Hall S Janelidze S Surova Y Cerebrospinal fluid concentrations of inflammatory markers in Parkinson's disease and atypical parkinsonian disorders. Sci Rep 2018;8:13276. 30185816 10.1038/s41598-018-31517-z PMC6125576

[24] Shamseer L Moher D Clarke M Preferred reporting items for systematic review and meta-analysis protocols (PRISMA-P) 2015: elaboration and explanation. BMJ 2015;350:g7647. 25555855 10.1136/bmj.g7647

[25] Stang A Critical evaluation of the Newcastle-Ottawa scale for the assessment of the quality of nonrandomized studies in meta-analyses. Eur J Epidemiol 2010;25:603–5. 20652370 10.1007/s10654-010-9491-z

